# Cumulative Genetic Risk for Asthma Contributes to Disease Severity in Children with Asthma living in Urban Environments

**DOI:** 10.1101/2025.09.08.25335346

**Published:** 2025-09-09

**Authors:** Matthew Dapas, William Wentworth-Sheilds, Emma E. Thompson, Rajesh Kumar, Elizabeth Lippner, Robert A. Wood, George T. O’Connor, Gurjit K. Khurana Hershey, Rebecca S. Gruchalla, Andrew H. Liu, Edward M. Zoratti, Leonard B. Bacharier, Stephanie Lovinsky-Desir, Michele A. Gill, William J. Sheehan, Shilpa J. Patel, Matthew C. Altman, James E. Gern, Cynthia M. Visness, Peter J. Gergen, Patrice M. Becker, Daniel J. Jackson, Carole Ober

**Affiliations:** 1. Division of Rheumatology, Department of Medicine; Center for Human Immunobiology; Center for Genetic Medicine; Northwestern University Feinberg School of Medicine, Chicago, IL; 2. Department of Human Genetics, University of Chicago, Chicago, IL; 3. Ann and Robert H. Lurie Children’s Hospital; Division of Allergy and Immunology, Department of Pediatrics, Northwestern University Feinberg School of Medicine, Chicago, IL; 4. Department of Pediatrics, Johns Hopkins University Medical Center, Baltimore, MD; 5. Pulmonary Center, Boston University School of Medicine, Boston, MA; 6. Division of Asthma Research, Cincinnati Children’s Hospital, Cincinnati, OH; 7. Department of Internal Medicine, University of Texas Southwestern Medical Center, Dallas, TX; 8. Children’s Hospital Colorado; Department of Allergy and Immunology, University of Colorado School of Medicine, Aurora, CO; 9. Departments of Medicine and Public Health Sciences, Henry Ford Health and Michigan State University College of Human Medicine, Detroit, MI; 10. Monroe Carell Jr. Children’s Hospital at Vanderbilt University Medical Center, Nashville, TN; 11. Department of Pediatrics and Environmental Health Sciences, Columbia University, New York, NY; 12. Department of Pediatrics, Washington University School of Medicine, St. Louis, MO; 13. Children’s National Hospital; Division of Allergy and Immunology, George Washington University School of Medicine and Health Sciences, Washington, DC; 14. Division of Emergency Medicine, Children’s National Hospital, Washington, DC; 15. Department of Allergy and Infectious Diseases, University of Washington, Seattle, WA; 16. Departments of Pediatrics and Medicine, University of Wisconsin School of Medicine and Public Health, Madison, WI; 17. Rho Inc., Durham, NC; 18. National Institute of Allergy and Infectious Diseases, Bethesda, MD

## Abstract

**Background:**

Childhood-onset asthma is highly heritable, with nearly 200 risk loci identified in genome-wide association studies. Aggregated polygenic risk scores can be used to quantify genetic predisposition to asthma, but their power to predict asthma severity in multi-ancestral groups has not been determined.

**Objective:**

Our aim was to examine the predictive power of biobank-derived asthma polygenic risk scores in children with asthma living in urban environments.

**Methods:**

We generated polygenic risk scores for asthma, derived from a large-scale genome-wide association meta-analysis, in four multi-ancestry asthma study cohorts of children living in urban environments. We assessed genetic predictions across different subphenotypes of asthma and tested for associations between genetic asthma risk and measures of asthma severity.

**Results:**

Genetic asthma prediction was significantly stronger for more symptomatic asthma phenotypes (P<0.001). Polygenic risk scores were significantly higher in difficult-to-control vs. easy-to-control asthma (P=0.02). Genetic risk was also significantly associated with more frequent exacerbations (P=0.03), higher blood eosinophil levels (P=0.01), and lower lung function (P<0.001).

**Conclusion:**

Cumulative genetic risk for asthma is associated with disease severity and exacerbation risk in children with asthma living in urban environments.

## INTRODUCTION

Children living in urban environments face an excess burden of asthma morbidity and mortality^1^. Urban environments are enriched for environmental exposures and sociodemographic factors that are associated with increased asthma prevalence and severity^2,3^. Genetics also contributes to the overall risk of developing asthma, as evidenced by nearly 200 risk loci uncovered in a large genome-wide association study (GWAS)^4^. Understanding how genetic risk contributes to asthma severity in children living in urban environments could lead to more accurate risk stratification and ultimately help guide personalized management strategies.

Genetic risk can be modeled using aggregated polygenic risk scores (PRSs). Many studies have developed predictive PRSs for asthma^5^, and phenome-wide association studies with asthma PRSs have suggested that genetic risk may correlate with asthma severity^5,6^. However, the relationship between cumulative genetic asthma risk and disease severity has not been fully explored^6^. Here, we generated PRSs for asthma in four multi-ancestry asthma study cohorts of children living in urban environments. We then assessed genetic predictions for different subphenotypes of asthma and tested for associations between genetic asthma risk and multiple measures of asthma severity.

## RESULTS

We utilized genome-wide genotype data from four multi-ancestry asthma cohorts of children living in urban environments (APIC^7^, URECA^8^, MUPPITS-1^9^, and MUPPITS-2^1^), as summarized in Table 1, including 1,108 children with childhood-onset asthma. The APIC (Asthma Phenotypes in Inner-City Children) study was an observational study in which different phenotypes of childhood-onset asthma (clusters A-E) were identified according to varying symptoms, biomarkers, and disease severity^7^. The URECA (Urban Environment and Childhood Asthma) study is a birth cohort enriched for family history of asthma or allergic disease^8^. The MUPPITS (Mechanisms Underlying Asthma Exacerbations Prevented and Persistent with Immune Based Therapy: A Systems Approach) studies investigated exacerbations in children with exacerbation-prone eosinophilic asthma^1,9^. Nearly all of the participants in these studies were parent-identified as Black or Hispanic, and the cohorts featured similar overall distributions of race/ethnicity (Table 1). Non-asthma population-based controls from the Consortium on Asthma in African-ancestry Populations in the Americas (CAAPA)^10^ were used as a comparator group (n=205). The distribution of ancestry PCs by cohort are shown in Figure S1.

Polygenic risk for asthma was modeled using PRS-CS^11^ on genome-wide association study summary statistics, as in our previous study^5^, from a large multi-ancestry genetic association meta-analysis from the Global Biobank Meta-analysis Initiative (GBMI; 153,763 cases and 1,647,022 controls)^4^. PRSs were derived from the intersect of genotyped single nucleotide variants (SNVs) across all genotyped cohorts (n=791,608 SNVs).

**Figure 1.**
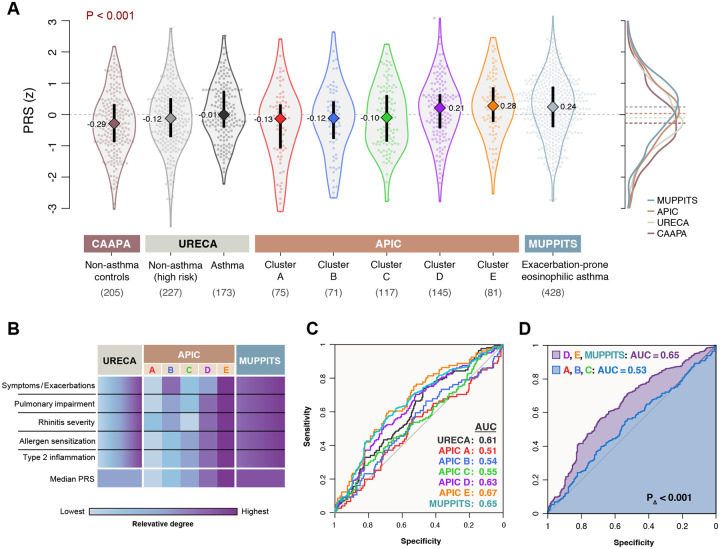
Genetic asthma prediction is stronger in more symptomatic phenotypes. A) Distributions and median values of asthma PRSs by genotyped cohort, including APIC’s previously defined sub-phenotypes (clusters A-E)^7^. B) Relative degrees of categorical asthma severity for each cohort. C) Receiver operating characteristic (ROC) curves with areas under the curve (AUC) for predicting asthma using the PRS for each cohort. D) Prediction of asthma in the combined moderate-to-severe asthma cohorts (APIC D, E & MUPPITS) was significantly more accurate than for the milder asthma subphenotypes (APIC A, B, C), combined.

PRS distributions differed significantly by cohort (P<0.001, Kruskal-Wallis), with higher median PRSs observed in cohorts characterized by more severe forms of asthma. (Figures 1A, 1B). Notably, the PRSs were significantly higher in children without asthma but enriched for family history of asthma than in population-based controls without asthma, likely owing to inherited disease risk (P=0.03, Wilcoxon). The PRS predicted asthma diagnosis more accurately in cohorts characterized by greater symptom burden, according to relative areas under the receiver operating characteristic curve (AUCs, Figure 1C). Overall, the PRS yielded significantly better prediction for moderate-severe asthma (AUC=0.65) than for the other asthma phenotypes (AUC=0.53; P_Δ_=<0.001, Figure 1D). These results demonstrate that genetic asthma prediction is stronger for more symptomatic phenotypes and further suggest that genetic risk correlates with increasing disease severity in children.

**Figure 2.**
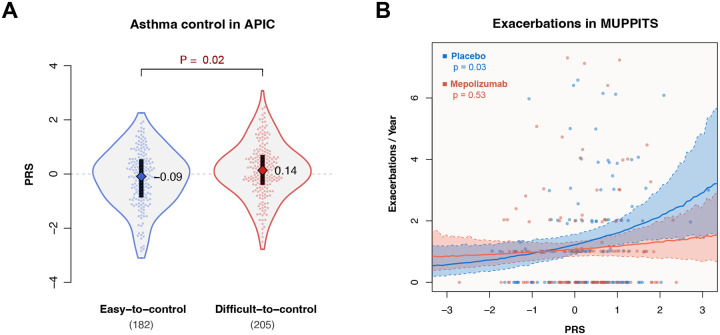
Polygenic risk is associated with asthma severity and exacerbation risk. A) Distributions and median values of asthma PRSs in APIC by required treatment step^12^. B) Exacerbation rate vs. PRSs in MUPPITS-2, split by treatment arm.

To more directly test whether polygenic asthma risk correlates with disease severity, we tested PRS associations with severity metrics within individual study cohorts. In APIC, PRSs were significantly higher in difficult-to-control vs. easy-to-control asthma^12^ (P=0.02; Figure 2A), indicating that children with more genetic risk alleles tended to require higher treatment levels to control their asthma. In the MUPPITS-2 trial, which investigated the effect of an anti-IL-5 inhibitor (mepolizumab) in children with exacerbation-prone eosinophilic asthma^1^, polygenic risk was significantly associated with higher exacerbation rates. However, the association was only observed in the placebo-treated group (P=0.03; Figure 2B). The correlation was not significant in the study arm treated with mepolizumab (P=0.53), suggesting that the biologic effectively reduced the genetic effect on the exacerbation rate.

**Figure 3.**
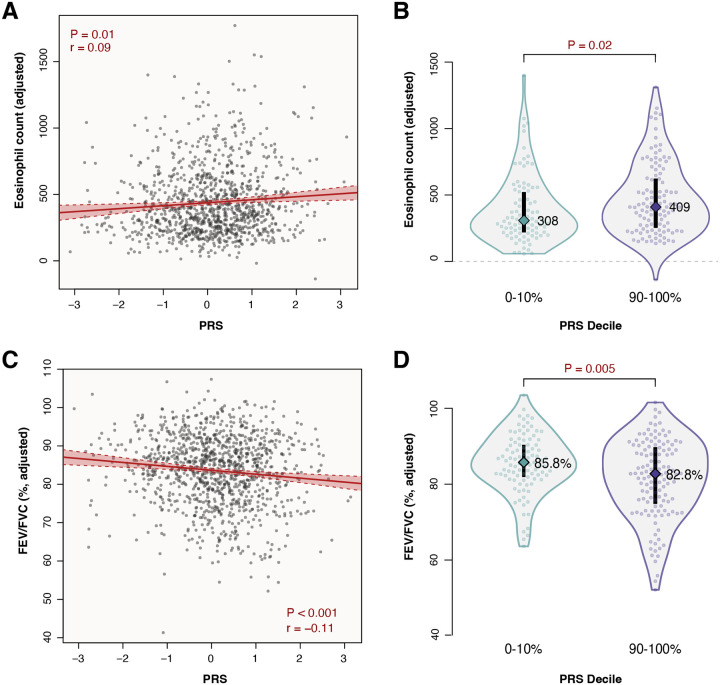
Polygenic risk is associated with quantitative biomarkers of asthma severity. A) Eosinophil count per μL, adjusted for age, sex, study, and ancestry, plotted against the PRS in a combined sample from APIC, URECA, and MUPPITS (n=1,062). The modeled correlation fit with 95% confidence interval is drawn in red. B) Distributions and median values of adjusted eosinophil counts in the lowest and highest PRS deciles. C) Adjusted FEV_1_/FVC plotted against the PRS in a combined sample from APIC, URECA, and MUPPITS (n=1,098). D) Distributions and median values of adjusted FEV_1_/FVC in the lowest and highest PRS deciles.

We next assessed associations between the PRS and quantitative biomarkers of asthma severity, adjusting for age, sex, study, and genetic ancestry (**Figure E1** in the Online Repository)^13^. In children with asthma across all cohorts, the PRS was significantly correlated with higher blood eosinophil levels (P=0.01; Figure 3A) and lower lung function (P<0.001; Figure 3C), as measured by the ratio of forced expiratory volume in one second to forced vital capacity (FEV_1_/FVC). Comparing the highest to lowest PRS deciles, these effects amounted to significant median differences of 101 eosinophils per μL (P=0.02; Figure 3B), and - 3.0% FEV_1_/FVC (P=0.005; Figure 3D). These findings further demonstrate that genetic asthma risk is correlated with quantitative measures relevant to asthma severity.

## DISCUSSION

Genetic disease risk depends on phenotype definitions, ancestries, and environmental exposures. The GBMI asthma GWAS that we used for modeling our PRS drew predominantly from European and East Asian adult cohorts and defined asthma according to specific International Classification of Diseases (ICD)-9 and ICD-10 codes in participant electronic health records^14^. Therefore, the asthma phenotypes, ancestries, and environments captured in the GBMI GWAS differ overall from the four cohorts studied here, which were limited to childhood-onset asthma cases in urban environments in the United States, consisting largely of Black and Hispanic populations, as well as from the CAAPA controls, which were African American. PRS predictive performance deteriorates across ancestries^15^, and we previously demonstrated how modeling genetic risk from different asthma GWASs yielded PRSs with different trait associations^5^. Furthermore, asthma cases in the GBMI GWAS consisted largely of adult-onset disease^4,5^, and the genetic architectures of childhood- and adult-onset asthma are partly distinct, with reported genetic correlations ranging from 0.67–0.78^16,17^. Although the GBMI meta-analysis is the largest and most diverse asthma GWAS conducted to date, and the GBMI-derived PRS predicted asthma in each of the four cohorts tested here with comparable accuracy to previous asthma PRS studies^5^, the PRS is not a perfect proxy for genetic asthma risk in these cohorts. Thus, the actual correlation between genetic asthma risk and disease severity is likely stronger than the effects we observed here.

Among patients with physician-diagnosed asthma according to ICD-10 codes, the vast majority have mild asthma, and the prevalence of severe asthma is less than 5%^18^. Asthma in the GBMI GWAS was primarily defined according to ICD codes in the constituent biobanks. Therefore, it is unlikely that the PRS correlations we observed with asthma severity are due to asthma in GBMI being disproportionately severe. Instead, our results indicate that the cumulative burden of genetic risk variants for asthma in general contributes to disease severity. This conclusion is supported by GWASs of moderate-to-severe asthma, which found that 88% of association signals that reached genome-wide significance had been previously reported in studies that predominantly assessed samples from patients with mild asthma^19,20^. Our findings expand on those from a population-based longitudinal study in European ancestry individuals, in which asthma cases with a higher polygenic burden among 17 risk variants were more likely to manifest atopy, airway hyperresponsiveness, and incompletely reversible airflow obstruction, and more often experienced hospitalizations for breathing problems^21^. Our results also align with another study that found polygenic risk for asthma was significantly associated with asthma exacerbations in European ancestry individuals^22^. Similar observations, where cumulative genetic risk from low-effect variants were associated with disease severity. have been reported in other complex diseases, as well, including cardiovascular disease^23^ and autoimmune disease^24^. Such genetic effects combine with those from rare variants^19^ and environmental exposures^25^ to contribute to disease severity through different pathways^2^.

In summary, the results of our study indicate that the cumulative effect of asthma-associated genetic variants not only increase disease risk but also contribute to disease severity. The effects observed in this study transcend ancestry, asthma phenotypes, and environments. Genetic risk models that are more closely aligned to demographic and clinical profiles of study populations could more precisely capture genetic influences on severity. Future studies should consider incorporating genetic risk for developing more comprehensive and accurate prediction models of asthma severity to inform strategies for clinical prevention, intervention, and management.

## METHODS

### Cohorts

We analyzed samples and phenotypes from several National Institutes of Allergy and Infectious Diseases (NIAID)-funded asthma studies conducted by the Inner-City Asthma Consortium (ICAC)^26^: the Asthma Phenotypes in the Inner City (APIC) study^7,12^, the Urban Environment and Childhood Asthma (URECA) birth cohort study^8^, and the Mechanisms Underlying Asthma Exacerbations Prevented and Persistent with Immune Based Therapy: A Systems Approach (MUPPITS 1 & 2) studies^1,9^. Non-asthma, population-based controls from the Consortium on Asthma among African-ancestry Populations in the Americas (CAAPA) study^10^ was used as a comparator group to assess the distribution of the polygenic risk score (PRS) in an unselected healthy population with similar ancestry to the APIC, URECA, and MUPPITS participants. Ethical approval for this work was granted by a central institutional review board, WGC IRB^27^.

#### APIC

APIC was a prospective, observational study in children with asthma age 6–17 years old from low-income areas (≥20% of residents below federal poverty level) of nine U.S. cities. Hierarchical clustering was performed on a large collection of baseline demographic, clinical, and environmental variables to identify different phenotypes of childhood-onset asthma. Five clusters were described (clusters A-E) according to varying symptoms, biomarkers, and disease severity^7^. APIC participants were also grouped based on required asthma controller treatment levels. Easy-to-control asthma was defined as requiring ≤100mcg/day of fluticasone at a majority of study visits, whereas difficult-to-control asthma was defined as requiring ≥500mcg/day^12^. APIC participants were genotyped using whole-genome sequencing (WGS) as previously described^13^.

#### URECA

URECA was a prospective, observational study of a birth cohort enriched for family history of asthma or allergic disease in four U.S. cities^8^. All families lived in neighborhoods where at least 20% of the population had incomes below the poverty level. Clinical measurements from age 10 were used when available; otherwise, the most recent measurement after age 5 were used. URECA participants were genotyped using WGS as previously described^13^.

#### MUPPITS 1 & 2

The MUPPITS studies investigated exacerbations in children with exacerbation-prone eosinophilic asthma who were living in urban environments across nine U.S. cities. Participants in MUPPITS were 6–17 years old, had blood eosinophils of ≥150 cells/μL, required at least twice-daily treatment with inhaled corticosteroids, and had at least two exacerbations treated with systemic corticosteroids in the previous year. All participants lived in census tracts where at least 10% of families had incomes below the poverty level^28^. The MUPPITS-1 study was a prospective, longitudinal study that compared transcriptome profiles in children who experienced cold symptoms between those that subsequently experienced an exacerbation and those that did not^9^. The MUPPITS-2 study was a randomized controlled trial to assess the safety and efficacy of mepolizumab for reducing exacerbations in this population^1^. Genotypes for MUPPITS 1 & 2 were determined in 461 subjects with available DNA using the Illumina Genome Diversity Array (GDA, 8v1.0, hg19) at the University of Chicago. Individual samples were removed if they met any of the following quality control (QC) criteria: genotyped vs. self-reported sex mismatches, unexpected sample duplication, >2% missing genotypes across all variants, or autosomal heterozygosity >5 standard deviations from the mean. Variants were excluded under the the following QC criteria: >4% missing genotypes across all samples or exact Hardy-Weinberg p-value <5×10^−8^. A total of 1,661,770 directly genotyped single-nucleotide variants remained following QC. Variants were lifted from human reference hg19 to hg38 using CrossMap^29^. Eagle^30^ was used for genotype phasing and minimac4^31^ was used for genotype imputation. Imputation was performed using the TOPMed reference panel (r2 version 1.0.0) of >100,000 whole genome sequences (human genome build 38) and the Michigan Imputation Server^32^. Following QC, 428 samples from MUPPITS 1 & 2 remained for analysis.

#### CAAPA

CAAPA^10^ performed WGS on approximately 1,000 individuals with African ancestry from 19 populations across the Americas and Caribbean as previously described^33^. The WGS data is available through dbGAP (accession phs001123.v2.p1). In this study, we utilized WGS data from CAAPA non-asthma control samples from among the African American populations (n=205). Variant QC followed the same criteria as applied to APIC and URECA WGS data^13^.

### Modeling Genetic Asthma Risk

Polygenic risk for asthma was modeled on association summary statistics from a large-scale, multi-ancestry genome-wide association study (GWAS) conducted as part of the Global Biobank Meta-analysis Initiative (GBMI). Summary statistics were available via https://www.globalbiobankmeta.org. The GWAS was a meta-analysis across 18 biobanks, consisting of 153,763 cases and 1,647,022 controls. Primary ancestry among asthma cases was 79.3% European, 12.1% East Asian, 3.3% African, 2.6% Admixed American, 2.6% Central and Southern Asian, and 0.01% Middle Eastern^4^. Asthma case and control statuses were assigned primarily according to “PheCodes” mapped from International Classification of Disease (ICD) codes, but several biobanks used self-reported data.

The polygenic risk model was generated using PRS-CS^11^ on the intersect of genotyped single nucleotide variants from GBMI and across all genotyped cohorts. PRS-CS applies a Bayesian regression framework with continuous shrinkage priors on variant effect sizes. The default half-Cauchy priors, α = 1 and β = 0.5, were used for determining local shrinkage parameters (ψj). We used the “PRS-CS-auto” algorithm to estimate the global shrinkage parameter (φ) directly from the GWAS summary statistics. For the Gibbs sampling used to derive the global shrinkage parameter and posterior variant effect sizes, we ran 10,000 Markov Chain Monte Carlo iterations with 5,000 burn-in steps. Each autosomal chromosome was modeled separately. We used the EUR linkage disequilibrium reference panel constructed by the PRS-CS developers using UK Biobank data. Posterior variant effects were aggregated into scores per individual using the Plink (v2.00a5.8LM AVX2 Intel, 21 Nov 2023) “score” function^34^, with missing genotypes imputed as the posterior effect size multiplied by the effect allele frequency. Cumulative PRSs were converted to Z scores (across all genotyped cohorts) for analysis.

### Ancestry estimation

Ancestry principal components (PCs) were calculated on the intersect of high-quality single-nucleotide variants genotyped in the asthma cohorts and several reference panels from the 1000 Genomes Project (1KG; n=156)^35^ and the Human Genome Diversity Project (HGDP; n=52)^36^, as in our previous study^13^. Ancestry PCs were calculated accounting for subject relatedness using PC-Air^37^ and PC-Relate^38^, with initial kinship estimates derived using KING^39^. Kinship was estimated in iterations until resultant PCs stabilized (n=3).

### Association Testing

Differences in PRS distributions between groups were assessed using Wilcoxon rank-sum tests for pairwise comparisons and Kruskal-Wallis tests for more than two groups. Statistical differences in receiver operating characteristic areas under the curve were evaluated using DeLong’s test. PRS effects on exacerbation rates in MUPPITS 2 were determined using negative binomial generalized linear models, adjusting for age, sex, and the first three PCs of global ancestry (**Figure E1**). PRS associations with quantitative traits across multiple studies were assessed using a two-stage approach^13,40,41^. First, traits were regressed on age, sex, study, and the first three ancestry PCs. Then, model residuals were rank-normalized and regressed again on the same covariates with the addition of the PRS as a predictor. Adjusted trait values for plotting PRS associations were derived by adding first-stage model intercepts to residuals.

## Supplementary Material

1

## Figures and Tables

**Table 1.** Demographic characteristics of childhood-onset asthma cohorts

Characteristic	All	APIC	URECA	MUPPITS 1 & 2
Number	1462	507	527	428
Age, years, median (range)	10 (6–17)	11 (6–17)	10 (7–10)	11 (6–17)
Female sex	665 (45%)	216 (43%)	261 (50%)	188 (44%)
*Race/Ethnicity*				
Black (non-Hispanic)	966 (66%)	318 (63%)	377 (72%)	269 (63%)
White (non-Hispanic)	23 (2%)	7 (1%)	7 (1%)	9 (2%)
Hispanic	378 (26%)	154 (30%)	104 (20%)	120 (28%)
Other/mixed	93 (6%)	26 (5%)	38 (7%)	29 (7%)
Unknown	4 (<1%)	2 (<1%)	1 (<1%)	1 (<1%)
*Site*				
Baltimore	233 (16%)	84 (17%)	149 (28%)	-
Boston	228 (16%)	65 (13%)	124 (24%)	39 (9%)
Chicago	97 (7%)	62 (12%)	-	35 (8%)
Cincinnati	84 (6%)	45 (9%)	-	39 (9%)
Dallas	86 (6%)	38 (7%)	-	48 (11%)
Denver	103 (7%)	59 (12%)	-	44 (10%)
Detroit	106 (7%)	50 (10%)	-	56 (13%)
New York	227 (16%)	65 (13%)	99 (19%)	63 (15%)
St. Louis	207 (14%)	-	155 (29%)	52 (12%)
Washington, D.C	91 (6%)	39 (8%)	-	52 (12%)
*Clinical traits*				
Asthma Diagnosis	1108 (75%)	507 (100%)	173 (33%)	428 (100%)
Eosinophils/μL, median (IQR)	300 (190–500)	300 (160–485.5)	200 (100–350)	400 (300–600)
FEV_1_/FVC, median (IQR)	0.80 (0.73–0.86)	0.79 (0.72–0.85)	0.84 (0.79–0.88)	0.76 (0.68–0.82)

APIC, Asthma Phenotypes in Inner-City Children; FEV1, Forced expiratory volume in one second; FVC, Forced vital capacity; IQR, interquartile range; MUPPITS, Mechanisms Underlying Asthma Exacerbations Prevented and Persistent with Immune Based Therapy: A Systems Approach; URECA, Urban Environment and Childhood Asthma

## Data Availability

Phenotype, genotype, GWAS summary statistics, and whole-genome sequencing files are available in dbGaP under accession phs002921 for the APIC and URECA studies and under accession phs004273 for the MUPPITS studies.
