# Supplementary material for: Cumulative Genetic Risk for Asthma Contributes to Disease Severity in Children with Asthma living in Urban Environments
Source: medRxiv. 2025 Sep 9:2025.09.08.25335346. Preprint. [Version 1] doi: 10.1101/2025.09.08.25335346 (PMC12440043; doi:10.1101/2025.09.08.25335346)
Supplement: 1 [file NIHPP2025.09.08.25335346V1-supplement-1.pdf]

medRxiv preprint doi: https://doi.org/10.1101/2025.09.08.25335346; this version posted September 9, 2025. The copyright holder for this preprint (which was not certified by peer review) is the author/funder, who has granted medRxiv a license to display the preprint in perpetuity.
It is made available under a CC-BY 4.0 International license .

198 **SUPPLEMENTAL FIGURES**

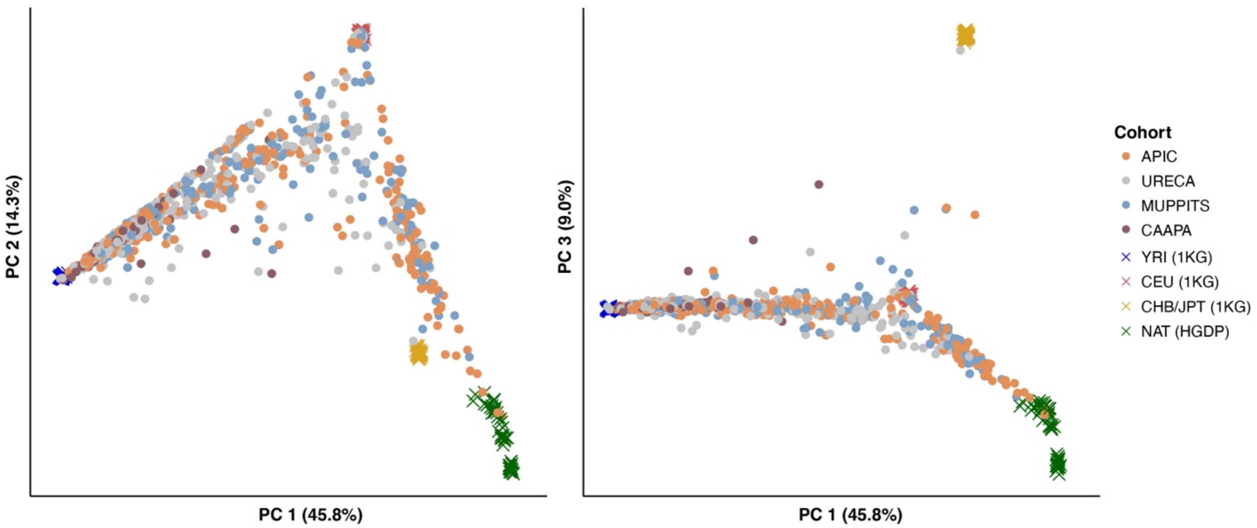

Figure S1. Principal components of ancestry by cohort. The top three principal components (PCs) of ancestry are plotted for sequenced APIC, URECA, MUPPITS, and CAAPA participants, colored by study, along with the four ancestry reference populations used for determining ancestry.

199
